# The Slower Antibody Response in Myelofibrosis Patients after Two Doses of mRNA SARS-CoV-2 Vaccine Calls for a Third Dose

**DOI:** 10.3390/biomedicines9101480

**Published:** 2021-10-15

**Authors:** Fabio Fiorino, Anna Sicuranza, Annalisa Ciabattini, Adele Santoni, Gabiria Pastore, Martina Simoncelli, Jacopo Polvere, Sara Galimberti, Stefano Auddino, Claudia Baratè, Francesca Montagnani, Vincenzo Sammartano, Monica Bocchia, Donata Medaglini

**Affiliations:** 1 Laboratory of Molecular Microbiology and Biotechnology, Department of Medical Biotechnologies, University of Siena, 53100 Siena, Italy; fiorino4@unisi.it (F.F.); annalisa.ciabattini@unisi.it (A.C.); gabiria.pastore@unisi.it (G.P.); jacopo.polvere@student.unisi.it (J.P.); stefano.auddino@student.unisi.it (S.A.); 2 Hematology Unit, Department of Medical Science, Surgery and Neuroscience, Azienda Ospedaliero Universitaria Senese, University of Siena, 53100 Siena, Italy; sicuranza4@unisi.it (A.S.); adele.santoni@gmail.com (A.S.); marti.simoncelli@gmail.com (M.S.); vincenzo.sammartano2@gmail.com (V.S.); 3 Section of Hematology, Department of Clinical and Experimental Medicine, University of Pisa, 56126 Pisa, Italy; sara.galimberti@med.unipi.it (S.G.); claudia.barate@gmail.com (C.B.); 4 Department of Medical Sciences, Infectious and Tropical Diseases Unit, Azienda Ospedaliero Universitaria Senese, University of Siena, 53100 Siena, Italy; francesca.montagnani@unisi.it; 5 Department of Medical Biotechnologies, University of Siena, 53100 Siena, Italy

**Keywords:** mRNA SARS-CoV-2 vaccination, myelofibrosis, ruxolitinib, COVID-19, antibody response, third booster dose

## Abstract

Immunization with mRNA SARS-CoV-2 vaccines has been highly recommended and prioritized in fragile subjects, including patients with myelofibrosis (MF). Available data on the vaccine immune response developed by MF patients and the impact of ruxolitinib treatment are still too fragmented to support an informed decision on a third dose for this category of subjects. Here, we show that 76% of MF patients develop spike-specific IgG after the second mRNA SARS-CoV-2 vaccine dose, but the response has a slower kinetics compared to healthy subjects, suggesting a reduced capability of their immune system to promptly react to vaccination. A reduced ACE2/RBD binding inhibition activity of spike-specific antibodies was also observed, especially in ruxolitinib-treated patients. Our results, showing slow kinetics of antibody responses in MF patients following vaccination with mRNA SARS-CoV-2 vaccines, support the need for a third vaccine dose.

## 1. Introduction

Onco-hematologic patients have paid a great price in terms of the COVID-19 disease caused by pandemic SARS-CoV-2 infection, with a mortality rate reaching 34% of infected patients [1,2]. Immunization with mRNA SARS-CoV-2 vaccines has been highly recommended and prioritized in fragile categories, including patients with hematologic malignancies (https://www.cdc.gov/coronavirus/2019-ncov/vaccines/recommendations/immuno.html, accessed on 8 October 2021). However, consolidated data regarding the efficacy of COVID-19 vaccines in this setting and the need for a third vaccine dose are still lacking. Patients with myelofibrosis (MF), a clonal hematopoiesis stem cell disorder belonging to the Philadelphia-negative myeloproliferative neoplasms (MPN), have a considerable higher risk of mortality after COVID-19 disease compared to the general population [1]. In addition, SARS-CoV-2 infection elicits an impaired antibody response in this subgroup of MPNs [3], similarly to other neoplastic hematologic disorders. In many MF patients, the myeloproliferative disease can be controlled by the JAK1/JAK2 inhibitor ruxolitinib, a powerful inhibitor of JAK-STAT signaling that deeply reduces inflammatory cytokine production and impairs, to some extent, cellular immune responses [4]. Ruxolitinib has also been successfully employed in dampening the cytokine storm responsible for fatal acute respiratory distress syndrome following severe COVID-19 infection, as documented by several studies [5,6]. On the other hand, the sudden interruption of ruxolitinib in MF COVID-19 infected patients has been followed by much higher death rate, probably due to the cytokine rebound following the withdrawal of the drug [7].

Based on these premises, it is of sure interest to investigate the immune response against SARS-CoV-2 elicited by vaccination in MF patients and the potential active role of ruxolitinib in influencing this response. To date, preliminary data of mRNA SARS-CoV-2 vaccination observed in small groups of MPN patients are still quite controversial. In fact, ruxolitinib was found to interfere negatively with the generation of the humoral immune response after a single dose of mRNA vaccines in 30 MPNs patients (13 with MF) as compared to healthy subjects [8], while in another report, ruxolitinib was shown not to impair the serological immune response measured after an unspecified period of time from receiving the second dose of BNT162b2 mRNA vaccine in 10 MF patients [9]. Different antibody responses have been reported among 42 MPN patients, with lower response to mRNA vaccines in patients with MF (10 subjects) compared to subjects affected by polycythemia vera and essential thrombocythemia [10]. On the contrary, another study conducted on 20 MPN subjects reported that patients with a diagnosis of MF (*n* = 9) had significantly higher post-first-dose anti-spike IgG and neutralizing antibody titers compared to patients with other MPN subtypes [11]. 

Given these still-fragmented and contradictory data, better understanding the immune response elicited by mRNA SARS-CoV-2 vaccines in MF patients with or without ruxolitinib treatment is extremely important in view of the potential need of a third dose.

Indeed, not enough is known about groups that might need a third vaccine dose, such as those with a compromised immune system [12]. The Centers for Disease Control and Prevention (CDC) recommends the third dose for immunocompromised subjects, including blood cancer patients under treatment (https://www.cdc.gov/coronavirus/2019-ncov/vaccines/recommendations/immuno.html, accessed on 8 October 2021), while the World Health Organization (WHO) called for a moratorium on boosters until at least the end of the year (https://www.who.int/director-general/speeches/detail/who-director-general-s-opening-remarks-at-the-media-briefing-on-covid-19, accessed on 8 September 2021; https://www.who.int/news/item/10-08-2021-interim-statement-on-covid-19-vaccine-booster-doses, accessed on 8 September 2021). Several countries have started or are in the process of starting the third dose administration in fragile subjects, despite the vaccine immune response of each category of fragile subjects have not been fully elucidated. 

Here, we prospectively profiled the spike-specific antibody response and the impact of the treatment with ruxolitinib in 42 consecutive MF patients, referring to our Hematology Units, at two time points (7 and 30 days) after the administration of the second dose of mRNA vaccine (Spikevax mRNA-1273 or Comirnaty BNT162b2), and in 40 healthy volunteers as control (HC).

## 2. Material and Methods

### 2.1. Study Design

A cohort of 42 patients with MF, vaccinated with mRNA SARS-CoV-2 vaccine (33 subjects with Spikevax mRNA-1273, 9 subjects with Comirnaty BNT162b2), and of 40 healthy, vaccinated volunteers (HC) as control was enrolled for the study. Patients with MF were treated (16/42) or not (26/42) with ruxolitinib. Plasma samples were collected at baseline (before vaccination), 7 and 30 days from the second dose of mRNA SARS-CoV-2 vaccine received 3–4 weeks after the first dose. The study was performed in compliance with all relevant ethical regulations, and the protocol was approved by the local Ethical Committee for Clinical experimentation of Regione Toscana Area Vasta Sud Est (CEASVE), protocol code 19479 PATOVAC_COV v1.0, approved on 15 Mar 2021. All participants provided written informed consent before participation in the study. Study participants were recruited at the Hematology Unit, Azienda Ospedaliera Universitaria Senese (Siena, Italy). 

### 2.2. ELISA

Maxisorp microtiter plates (Nunc, Denmark) were coated with recombinant SARS-CoV-2 full spike protein (S1 + S2 ECD, Sino Biological), with 50 μL per well of 1 μg/mL protein solution in PBS (Sigma-Aldrich, St. Louis, MO, USA), and left overnight at 4 °C. Plates were blocked at room temperature (RT) for 1 h with 200 μL of 5% skimmed milk powder (AppliChem, Darmstadt, Germany), 0.05% Tween 20, 1 × PBS. All plasma samples, heated at 56 °C for 1 h, were added and titrated in two-fold dilution in duplicate in 3% skimmed milk powder, 0.05% Tween 20 (Sigma-Aldrich)–, 1 × PBS (diluent buffer) and incubated 1 h at RT. Anti-human horseradish peroxidase (HRP)-conjugated antibody for IgG (diluted 1:6000; Southern Biotechnology, Birmingham, AL, USA) was added in diluent buffer for 1 h at RT. Plates were developed with 3,3′,5,5′-Tetramethylbenzidine (TMB) substrate (Thermo Fisher Scientific, Waltham, MA, USA) for 10 min at RT, followed by addition of 1M stop solution. Absorbance at 450 nm was measured on Multiskan FC Microplate Photometer (Thermo Fisher Scientific). A WHO international positive control (plasma from vaccinated donor diluted 1:5000; NIBSC) and negative control (plasma from unvaccinated donor diluted 1:20, NIBSC) were added in duplicate to each plate as internal control for assay reproducibility. Antibody end point titers were expressed as the reciprocal of the sample dilution, reporting double the background OD value.

### 2.3. ACE2/RBD Binding Inhibition Assay

ACE2/RBD binding inhibition was tested with a SARS-CoV 2 surrogate virus neutralization test (sVNT) kit (cPass™ SARS-CoV-2 Neutralization Antibody Detection Kit, Genscript, Piscataway, NJ, USA) according to the manufacturer protocol. Briefly, plasma samples, positive and negative controls were diluted 1:10 in dilution buffer, mixed 1:1 with HRP-RBD buffer and incubated for 30 min at 37 °C. An amount of 100 µL of each mixture was added to each well of an ACE2-coated 96-well flat-bottom plate and incubated for 15 min at 37 °C. The plate was washed four times with wash solution and tapped dry. An amount of 100 µL of TMB solution was added to each well and the plate was developed for 15 min at RT). After that, the reaction was quenched, adding 50 µL of the stop solution to each well, and the OD at 450 nm was instantly read with Multiskan FC Microplate Photometer (Thermo Fisher Scientific). Results of the ACE2/RBD inhibition assay are expressed as follows: percentage inhibition = (1 − sample OD value/negative control OD value) × 100. Inhibition values ≥30% are regarded as positive results, and values <30% as negative results, as established by Tan at al. [13] and indicated by the manufacturer.

### 2.4. Statistical Analysis

The Kruskal–Wallis test, followed by Dunn’s post-test for multiple comparisons, was used to assess the statistical differences of ELISA titers and ACE2/RBD binding inhibition percentages among different groups of subjects for each time point post vaccination. A *p*-value ≤ 0.05 was considered significant. Analyses were performed using GraphPad Prism v9 (GraphPad Software, San Diego, CA, USA). Multiple linear regression analyses were performed to create models for the prediction of vaccine response in patients based on their clinical data. Clinical information (treatment with ruxolitinib, age, lymphocytes pre-vaccination, gender, disease, centimeters of spleen below costal margin, IPSS score, age at diagnosis, LDH) was used as predictor variables and a model for each response variable (log-transformed IgG titers and ACE2/RBD binding inhibition, both 7 and 30 days after the second vaccine dose) was fitted. The multiple linear regression analyses were carried out in R platform (v4.1.1; R Fundation for Statistical Computing, Vienna, Austria). 

## 3. Results and Discussion

The antibody response after two doses of mRNA SARS-CoV-2 vaccine was studied in a cohort of 42 MF patients and 40 healthy volunteers as controls (HC). A total of 16/42 (38%) MF patients were on ruxolitinib at the time of vaccination, while 26/42 (62%) were receiving hydroxyurea or supportive therapy only. The clinical characteristics of MF patients are outlined in Table 1. 

**Figure 1:**
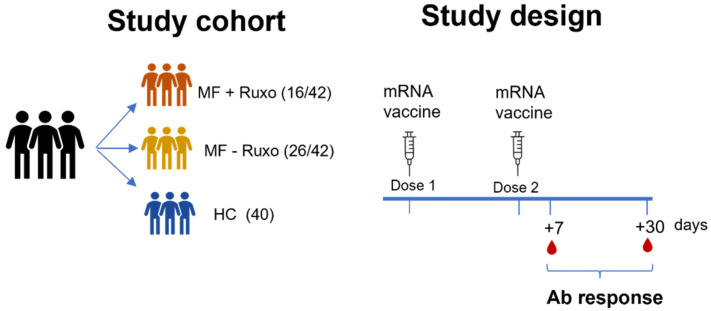
Schematic representation of the study cohort and design. Myelofibrosis (MF) patients treated or not with ruxolitinib (MF + ruxo or MF-ruxo) and healthy controls (HC) were immunized with two doses of mRNA vaccine anti SARS-CoV-2. Antibody (Ab) response was assessed 7 and 30 days after the second vaccine dose.

Patients received the second dose of mRNA anti-SARS-Cov-2 vaccine 3–4 weeks following the first dose, and plasma samples were collected 7 and 30 days later (Figure 1). Day 7 after the second dose is an early time point, corresponding to the antibody peak in healthy subjects [14], and is useful for evaluating the rapid increase in the recall response, while day 30 is a later time point useful to monitor the trend and the persistence of the humoral response. 

Plasma samples were tested for IgG against the SARS-CoV-2 spike protein [14] by ELISA and for ACE-2/RDB binding inhibition activity using a SARS-CoV-2 surrogate virus neutralization test (sVNT).

**Figure 2:**
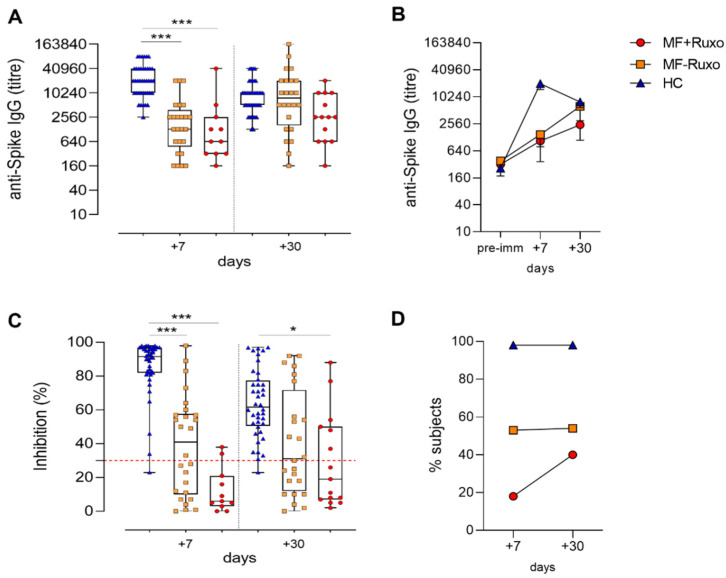
Humoral immune response against SARS-CoV-2 in MF patients post-second dose of mRNA vaccination. (**A**) IgG titers specific for the spike protein assessed by ELISA in plasma of patients with myelofibrosis (MF) patients, treated or not with ruxolitinib (MF + Ruxo or MF-Ruxo), and healthy controls (HC), at different time points after the second vaccine dose. Antibody end point titers were expressed as the reciprocal of the sample dilution, reporting double the background OD value. (**B**) Time course of spike-specific IgG in plasma collected at the baseline (pre-immune) and 7 and 30 days from the second dose of mRNA SARS-CoV-2 vaccine. (**C**) ACE2/RBD binding inhibition activity tested in plasma using a SARS-CoV-2 surrogate virus neutralization test. Results are expressed as ACE2/RBD binding inhibition percentage with box and whiskers diagram showing all subject values. Inhibition values >30% are regarded as positive results according to the manufacturer indication. (**D**) Percentage of subjects developing ACE2/RBD inhibition positive values (>30%) in the different groups. Kruskal–Wallis test, followed by Dunn’s post-test for multiple comparisons, was used for assessing statistical differences between groups. * *p* ≤ 0.05; *** *p* ≤ 0.001. Healthy control (HC), myelofibrosis (MF), ruxolitinib (Ruxo).

Seven days after the booster dose, the geometric mean tire (GMT) of anti-spike specific IgG in MF patients was significantly lower compared to HC, regardless of ruxolitinib treatment (GMT of 20180, 1470 and 1060 in HC, MF-Ruxo and MF + Ruxo, respectively; *p* < 0.001; Figure 2A). Nevertheless, 30 days after the second dose, anti-spike IgG increased significantly in MF patients, reaching antibody titers similar to the ones observed in HC, especially in the group without ruxolitinib treatment (GMT of 7896, 6267 and 2436 in HC, MF-Ruxo, MF + Ruxo, respectively; Figure 2A,B). When MF patients were analyzed according to the ruxolitinib treatment, the 68% of treated and 81% of untreated patients developed a spike-specific plasma IgG response, showing a slightly lower response in ruxolitinib-treated subjects. 

While the spike-specific IgG titers in HC were slightly declining a month after the booster dose (Figure 2B), as recently reported [14,15,16,17], the antibodies elicited in MF patients were still increasing, thus showing a different kinetic of the secondary response, with a slower development of the humoral response in MF patients. The antibody response dynamic observed in these subjects suggests a lower immunostimulating effect elicited by the first vaccine dose, as it was reported by two recent publications [8,10]. In particular, Pimpinelli et al. observed that MF subjects developed a lower humoral response compared to MPN patients with an undetectable primary immune response [10]. 

ACE2-RBD binding inhibition, a surrogate SARS-CoV-2 neutralization test that qualitatively detects antibodies capable of inhibiting the interaction between the receptor binding domain (RBD) of the viral spike glycoprotein and the angiotensin-converting enzyme 2 (ACE2) protein [13,18], was evaluated in plasma collected 7 and 30 days after the second vaccine dose (Figure 2C). The percentage of healthy subjects that developed a positive inhibition activity (>30%) 7 days after the booster dose was around 98% (39/40), and it was stably maintained at day 30 (Figure 2D). Among MF patients, a positive inhibition activity was observed in 14/26 (54%) subjects without ruxolitinib treatment at both time points, while in ruxolitinib-treated MF patients, the percentage of subjects developing antibodies with positive inhibitory activity was 18% (2/11) at day 7 and 40% (6/15) one month after the second vaccine dose (Figure 2D). In MF patients, expecially under ruxolitinib treatement, was observed a reduced ACE2-RBD binding inhibition activity of plasma antibodies compared to HC, even though the percentage of ruxolitinib-treated patients with a positive value increased over time. 

Multiple linear regression models were fitted on clinical parameters (treatment with ruxolitinib, age, lymphocytes pre-vaccination, gender, disease, centimeters of spleen below costal margin, IPSS score, age at diagnosis, LDH) for the prediction of the antibody response and their ACE2-RBD binding inhibition activity. None of the clinical parameters could be associated with the prediction of humoral response due to the lack of models with a significant *p*-value of the F-statistic.

Taken together, these data demonstrate that 76% of MF patients underwent seroconversion (calculated according to WHO guidelines https://www.who.int/csr/resources/publications/WHO_CDS_CSR_ARO_2004_1.pdf, accessed on 8 September 2021) [19] (Table 2) after two vaccine doses, but the development of the secondary antibody response in these patients showed a slower evolution compared to HC, in contrast with the characteristics of rapidity and intensity typically associated with secondary responses to booster doses, suggesting a reduced capability of their immune system to react to vaccination. This observation is particularly relevant for defining the vaccination schedule more fitting for these patients, suggesting a possible positive role for a third vaccine dose. 

Our results, obtained in a not negligible number of MF patients prospectively studied after two doses of mRNA vaccinations, shed a light on the controversial preliminary observations [8,9,10,11]. The slower antibody response observed in MF patients, regardless of ruxolitinib treatment, may explain the apparent impairment of anti-spike and neutralizing antibodies production claimed by Guglielmelli et al. in MPN patients on ruxolitinib [8] and measured after only one dose of mRNA vaccines. Given our results, the JAK1/2 inhibitor treatment slightly reduces the spike-specific antibody titers elicited by two vaccine doses (Figure 2B) but impacts their functionality, as observed by the reduced percentage of patients with a positive ACE2-RBD binding inhibition activity (Figure 2D). Notably, none of the 42 MF patients have developed COVID-19 infection after the vaccination so far. Regarding this, it should be taken into consideration that MF patients per se experience a much higher infection rate than other MPN patients [20]. Ongoing studies focused on the detection of spike-specific B memory cells will further shape both the natural and vaccine-mediated anti-COVID response in this subset of fragile patients and will better clarify the grade and persistence of the immune response against SARS-CoV-2 we may expect in mRNA-vaccinated MF patients. 

## 4. Conclusions

Our data show that MF patients, after two doses of mRNA SARS-CoV-2 vaccine, develop a humoral response with a slower kinetic compared to healthy subjects, and that a reduced antibody inhibition binding activity is observed, especially with ruxolitinib treatment. The antibody response dynamic observed in these subjects, suggesting a reduced capability of their immune system to promptly react to vaccination, calls for a third vaccine dose. Our results, therefore, contribute to answer the open question on the induction of the antibody responses in MF patients following vaccination with COVID-19 mRNA vaccines and on the impact of ruxolitinib treatment. This knowledge is of critical importance to assess the need for repeated booster doses of SARS-CoV-2 mRNA vaccines and guide vaccination policies tailored for MF patients, making the case for a third vaccine dose administration.

## Figures and Tables

**Table 1:** Clinical characteristics of MF patients included in the study. PV = polycythemia vera; ET= essential thrombocythemia.

Patients’ Characteristics	Whole Cohort (*n* = 42)	Ruxolitinib (*n* = 16)	No Ruxolitinib (*n* = 26)
Median age at diagnosis (range)	67 years (31–85)	63.5 years (44–85)	68.5 years (31–81)
Sex			
Male	21 (50%)	9 (56%)	12 (46%)
Female	21 (50%)	7 (44%)	14 (54%)
Disease			
Primary MF	21/42 (50%)	4/21 (19%)	17/21 (81%)
Post-PV	13/42 (31%)	9/13 (69%)	4/13 (31%)
Post-ET	8/42 (19%)	3/8 (37.5%)	5/8 (62.5%)
IPSS SCORE			
LOW	11/42 (26.2%)	4/11 (36%)	7/11 (64%)
INT—1	15/42 (35.8%)	6/15 (40%)	9/15 (60%)
INT—2	8/42 (19%)	4/8 (50%)	4/8 (50%)
HIGH	8/42 (19%)	4/8 (50%)	4/8 (50%)
Driver mutation			
JAK2	29/42 (69.1%)	15/29 (51.7%)	14/29 (48.3%)
CALR	9/42 (21.4%)	1/9 (1.1%)	8/9 (98.9%)
MPL	1/42 (2.4%)	0/1 (0%)	1/1 (100%)
Triple negative	3/42 (7.1%)	0/3 (0%)	3/3 (100%)
Time of exposition to ruxolitinib (range)		26 months (4–48 )	
Spleen below costal margin, median (range)	3 cm(0–20)	3 cm (0–20)	3.5 cm (0–20)
Hemoglobin g/dL, median (range)	11.8 (6.2–15.5)	11.6 (8.5–15.5)	12.4 (6.2–15.3)
Platelets × 10^3^/µL, median (range)	306 (19–789)	204 (19–724)	362 (36–789)
WBC × 10^3^/µL, median (range)	7.39 (2.7–37.15)	9.47 (3.5–37.15)	6.7 (2.7–25.27)
Lymphocytes × 10^3^/µL, median (range)	1.55 (0.12–7.4)	1.49 (0.51–7.4)	1.6 (0.12–3.03)
Total protein g/dL, median (range)	6.9 (5.8–8.5)	7.05 (5.9–8.5)	6.85 (5.8–7.8)
γ-Globulins (%), median (range)	13 (5.8–23.9)	13.2 (9–23.9)	12.9 (5.8–22)
LDH U/L, median (range)	415 (172–1430)	377 (209–1019)	498 (172–1430)

**Abbreviations:** INT-1, Intermediate-1; INT-2, Intermediate-2; JAK2, Janus Kinase 2; CALR, Calreticulin; MPL, thrombopoietin receptor gene.

**Table 2:** Number and percentage (in bracket) of patients with seroconversion and binding inhibition activity detected within MF patients (MF + Ruxo and MF-Ruxo) and HC subjects.

Subjects	Seroconversion	Binding Inhibition Activity of Seroconverted Subjects
Total MF	32/42 (76%)	22/42 (52%)
MF + Ruxo	11/16 (68%)	6/16 (37%)
MF − Ruxo	21/26 (81%)	16/26 (61%)
HC	40/40 (100%)	39/40 (98%)

## Data Availability

Data available upon request.
